# Breast Cancer Screening Practices in a Tertiary Care Center in the State of Qatar: A Cross-Sectional Survey

**DOI:** 10.2147/BCTT.S285210

**Published:** 2021-01-08

**Authors:** Jessiya Veliyankodan Parambil, Mostafa Najim, Mohamed Mahmoud, Ibrahim Yusuf Abubeker, Anand Kartha, Francois Calaud, Ahmed Al-Mohamed, Dabia Al-Mohannadi, Prem Chandra, Mohamed A Yassin

**Affiliations:** 1 Department of Internal Medicine, Hamad General Hospital, Hamad Medical Corporation, Doha, Qatar; 2 Department of Internal Medicine, Brown University, Providence, Rhode Island, USA; 3 Department of Medical Oncology, National Centre for Cancer Care and Research, Hamad Medical Corporation, Doha, Qatar; 4 Medical Research Center, Hamad Medical Corporation, Doha, Qatar

**Keywords:** breast cancer, screening, residency training program, physicians, Middle East

## Abstract

**Introduction:**

Breast cancer is the most common cancer in females. In Qatar, mortality related to breast cancer came in third after lung cancer and leukemia. In this study, we aim to comprehensively evaluate the rate of internal medicine residents and faculty compliance with breast cancer screening in Hamad Medical Corporation (Doha, Qatar), as well as to identify barriers and facilitators that could potentially augment changes to enhance physician-led cancer screening.

**Methods:**

A cross-sectional web-based survey was distributed among internal medicine physicians between December 2018 and March 2019 at a tertiary medical centre. It focused on the knowledge, attitude, and practice of physicians regarding breast cancer screening guidelines and explored potential barriers and proposed solutions. Chi-square and *t*-test statistics were used to draw conclusions where appropriate.

**Results:**

A total of 158 physicians responded to the survey, with a response rate of 61%. 75.9% were postgraduate trainees. Around three-quarters of the physicians mentioned that they would recommend breast cancer screening for their age-appropriate average-risk patients. There was a statistically significant difference between the trainees, consultants, and specialists regarding the modality of choice, where the majority of the trainees opted mammogram every 2 or 3 years while 44.4% of the consultants indicated yearly self-breast exam (*p*<0.001). The percentage of survey participants who rarely to never offer breast cancer screening in the outpatient settings was 37.8%. Unclear pathway (40%) and lack of time in clinic/ward rounds (26.5%) were the major reported barriers for cancer screening.

**Conclusion:**

In the current era of personalized medicine, physicians should be more oriented to local guidelines to provide optimal care to their patients. While the attitude towards breast cancer screening is positive, the overall compliance with the national recommendations is sub-optimal. Further initiatives and intervention programs are required to promote the breast cancer screening in Qatar.

## Introduction

Breast cancer is a major health concern. It is the most common cancer among females and the second most common cancer worldwide.^1^ The WHO estimates that 2.1 million women are diagnosed with breast cancer every year. Approximately 627,000 women die annually from breast cancer, accounting for 15% of all cancer deaths.^2^ The data available from the Gulf cooperation council (GCC) countries do not vary much from the international data as breast cancer remains the most common cancer among females.^3^ Focusing on the state of Qatar, which is one of the six members of GCC countries, breast cancer constitutes 15% of newly diagnosed cancer patients. In females, this equates to around 38% of all new cancer cases. The mortality related to breast cancer came in third after lung cancer and leukemia.^4^

The high mortality rate in breast cancer is attributable to delayed diagnosis. Recent data showed that breast cancer mortality is declining in high-income countries.^5^ It is still debatable whether this is related to the cancer screening programs that decrease the number of advanced-stage diseases that carry poor prognosis or the development of new breast cancer therapies. Nevertheless, breast cancer screening with mammograms remains an important tool, and it can decrease the overall mortality by almost 20%.^6^

It has been established that the knowledge and awareness of breast cancer screening programs are relatively low among females in Qatar.^7^ Among the various factors, physician’s recommendation is the most important variable that influences the participation in cancer screening programs. In this study, we aimed to evaluate physicians’ compliance with breast cancer screening in Hamad Medical Corporation (HMC) and to identify barriers and facilitators that could potentially augment changes that could enhance physician-led cancer screening.

## Study Methodology

**Figure 1:**
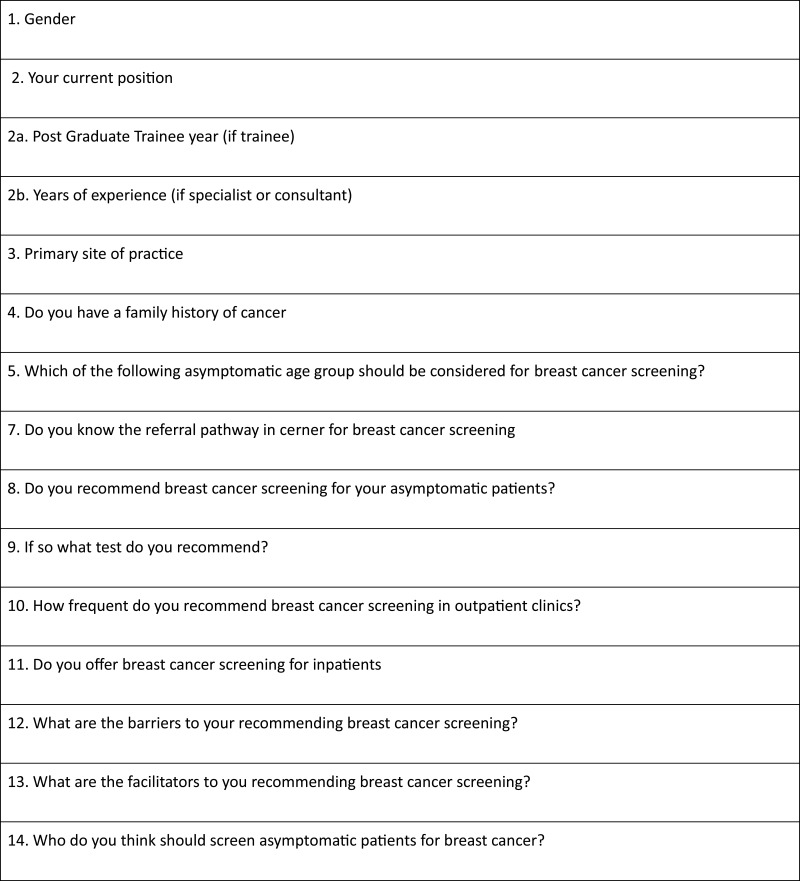
Breast Cancer screening survey questionnaire.

This survey is a cross-sectional multicenter observational study that aimed to evaluate physicians’ practice over a four-month period (December 2018–March 2019) at HMC. The constituent institutions were comprised of tertiary and specialized secondary health-care centers in the state of Qatar. The respondents were postgraduate trainees of the Internal Medicine Residency Program (IMRP) and faculty of the internal medicine department. A web-based standardized questionnaire [Figure 1] was administered to the participants via the corporate website with a pre-specified respondent target of at least 60% to enable reliable and dependable inferences to be made. Questions were designed to follow the Walsh and McPhee Systems Model of Clinical Preventive Care.^8^ The questionnaire structure was based on the study “Barriers to and Facilitators to Physician Recommendation of Colorectal Cancer Screening” by Guerra et al,^9^ with adjustment in the questions to fit our system in HMC. The responses were recorded in a Microsoft Excel database. The survey included questions on the knowledge and practice of breast cancer screening and discriminants to identify its barriers and facilitators. The questionnaire explored physicians’ knowledge of national and international breast cancer screening guidelines, as there is a significant difference in Qatar’s breast cancer incidence compared to the international population.

## Statistical Analyses

For categorical variables, frequencies and percentages were reported. Chi-square test or Fisher’s exact test was used where appropriate (n<5 or n=0). Yates Correction was used because both Pearson’s chi-square test and McNemar’s chi-square test are biased upwards for a 2 x 2 contingency table. All analyses were carried out using IBM^®^ SPSS^®^ Statistics V26.

## Results

Questionnaires were completed by 171 physicians. They were collected over a period of four months with a response rate of 61%. The majority of the respondents were postgraduate trainees (p = <0.001). Two-thirds of faculty members (consultants and specialists) reported clinical experience of more than 10 years, as shown in (Table 1, Table 3, Table 4, Table 5, Table 6).

**Table 1:** Demographic Characteristics of Physicians Responded to the Survey

Characteristics	Frequency (Total n=171)	Percentage
Male	118	69
Female	53	31
Postgraduate trainee	129	75.9
Specialist	9	5.3
Consultants	33	18.8
Postgraduate trainee year:		
PGY^a^1/2	59	45.7
PGY 3/4	68	52.7
PGY 5–7	2	1.6
Years of experience for faculty		
Up to 5	2	0.47
5–10	9	21.43
More than 10	31	73.8
Family history of cancer	54	31.8

**Note:** ^a^Postgraduate year.

### The Reported Practice of Cancer Screening

Out of a total of 171 physicians who participated in the survey, the majority (75.4%) mentioned that they would recommend breast cancer screening for their asymptomatic patients. There was a statistically significant difference in the proportion of male and female respondents in our study cohort (confidence interval [CI] 0.61–1.0; P = <0.001). Trainees recommend breast cancer screening more than the faculty members (p=0.026), and senior trainees do more than juniors (p=0.041). When patients were stratified into four age groups (40–60 years, 45–69 years, 50–75 years, and 55–80 years), 50% of study respondents recommended breast cancer screening for asymptomatic individuals aged between 40 and 60 years while 24.4% chose 50–75 years and 23.8% selected 45–69 years as the appropriate age for breast cancer screening. There was no statistically significant difference in recommending breast cancer screening among trainees, faculty members, training/experience levels, or the site of practice. Similarly, having a family history of cancer did not influence the physician’s decision to recommend cancer screening for asymptomatic individuals. Only 54.9% of survey respondents were aware of the electronic medical records platform (Cerner) for breast cancer screening. However, senior trainees tend to know the pathway better than the junior ones (p= 0.047).

Mammogram recommendations at two (32.4%) and three (31.1%) year intervals were the preferred choices for breast cancer screening. A self-breast exam was suggested by 16.2% of the survey participants. When stratifying the participant according to their positions, there was a statistically significant difference between the trainees, consultants, and specialists regarding the modality of choice for screening. The majority of the trainees opted mammogram every 2 or 3 years, while 44.4% of the consultants recommended an annual self-breast exam (*p*<0.001). There was no significant difference in the choice of tests among the different trainee levels. The percentage of survey participants who rarely offer breast cancer screening to their age-appropriate average-risk patients along with those who never offer the screening in the outpatient settings, was 37.8%. The tendency to offer outpatient asymptomatic breast cancer screening was not influenced by the gender of the respondents (Kendall Tau β = −0.45, P = 0.4), but senior trainees more frequently make the recommendations compared to juniors (p=0.049). For inpatient settings, the proportion of participants who recommend breast cancer screening for their patients was even lower. The percentage of survey participants who always offer breast cancer screening to their age-appropriate average-risk patients, along with those who often offer the screening, was 15.6%.

On the other hand, the percentage of physicians who rarely offer breast cancer screening, along with those who never offer the screening, was 63.2%. The position of the physician, years of experience, training level, or site of practice did not influence their cancer screening recommendation for inpatients. For details, please refer to Tables 2–7.

**Table 2:** The Reported Practice of Cancer Screening

	Frequency	Percentage
Asymptomatic age group considered for breast cancer screening?		
40–60	82	50.0
45–69	39	23.8
50–75	40	24.4
55–80	3	1.8
Do you recommend breast cancer screening for your asymptomatic patients?		
Yes	119	75.4
No	41	24.6
Do you know the referral pathway in Cerner for breast cancer screening?		
Yes	90	54.9
No	74	45.1
Recommended test for screening		
Self-breast exam/physical exam yearly	24	16.2
Mammogram yearly	29	19.6
Mammogram every 2 years	48	32.4
Mammogram every 3 years	46	31.1
MRI breast yearly	1	0.7
How frequent do you recommend breast cancer screening in outpatient clinics?		
Always	12	7.5
Often	30	18.9
Sometimes	39	24.5
Rarely	47	29.6
Never	13	8.2
Do not have clinic	18	11.3
Do you offer breast cancer screening for inpatients		
Always	8	5.0
Often	17	10.6
Sometimes	34	21.3
Rarely	67	41.9
Never	34	21.3

**Table 3:** Response to Do You Recommend Breast Cancer Screening for Asymptomatic Patients

	Yes	No	P-value
Your current position			
Trainee	86	35	0.026
Specialist	5	4	
Consultant	26	2	
Postgraduate trainee year			
PGY^a^ 1/2	41	15	0.554
PGY 3/4	43	20	
PGY 5–7	2	0	
Years of experience			
2–5	2	1	0.041
5–10	4	3	
more than 10	27	2	
The primary site of practice			
Hamad General H^b^	107	39	0.442
Al Wakra H	4	0	
Al Khor H	8	2	

**Notes:** ^a^Postgraduate trainee year ^b^Hospital.

**Table 4:** Age Group to Which Breast Cancer Screening Should Be Recommended

Categorical	40–60	45–69	50–75	55–80	P-value
Your current position					
Trainee	54	32	35	3	0.109
Specialist	5	2	2	0	
Consultant	22	4	3	0	
Postgraduate trainee year					
PGY^a^ 1/2	26	12	17	1	0.896
PGY 3/4	27	19	18	2	
PGY 5–7	1	1	0	0	
Years of experience					
2–5	2	0	1	3	0.064
5–10	4	4	0	8	
More than 10	22	3	4	29	
Primary site of practice					
Hamad General H^b^	73	37	36	3	0.945
Al Wakra H	3	1	1	0	
Al Khor H	6	1	3	0	

**Notes:** ^a^Postgraduate trainee year ^b^Hospital.

**Table 5:** The Test Offered for Breast Cancer Screening

	Physical Exam Yearly	Mammogram Yearly	Mammogram Every 2 Years	Mammogram Every 3 Years	MRI Breast Yearly	P-value
Your current position						
Trainee	9	21	41	39	0	<0.001
Specialist	3	2	1	3	0	
Consultant	12	5	5	4	1	
Postgraduate trainee year						
PGY^a^ 1/2	3	9	23	18	0	0.786
PGY3/4	6	12	17	20	0	
PGY5-7	0	0	1	1	0	
Years of experience						
2–5	1	0	0	1	0	0.643
5–10	2	3	2	0	0	
more than 10	12	5	5	6	1	
Primary site of practice						
Hamad General H^b^	20	26	47	40	1	0.693
Al Wakra H	1	1	0	2	0	
Al Khor H	3	2	1	4	0	

**Notes:** ^a^Postgraduate trainee year ^b^Hospital.

**Table 6:** Response to How Often Physician Offer Breast Cancer Screening in Their Clinics

	Always	Often	Sometimes	Rarely	Never	I Do Not Have a Clinic	P-value
Your current position							
Trainee	8	22	29	40	11	11	0.796
Specialist	1	1	2	2	1	2	
Consultant	3	7	7	5	1	4	
Postgraduate trainee year							
PGY^a^ 1/2	5	10	12	14	6	9	0.049
PGY 3/4	2	11	17	26	5	2	
PGY 5–7	1	1	0	0	0	0	
Years of experience							
2–5	0	0	1	0	1	0	0.135
5–10	1	2	1	2	1	0	
more than 10	3	6	8	5	0	7	
The primary site of practice							
Hamad General H^b^	12	26	35	42	12	18	0.776
Al Wakra H	0	1	1	1	1	0	
Al Khor H	0	3	3	4	0	0	

**Notes:** ^a^Postgraduate trainee year ^b^Hospital.

**Table 7:** Response to How Often Physician Offers Breast Cancer Screening for Inpatients

	Always	Often	Sometimes	Rarely	Never	P-value
Your current position						
Trainee	4	14	24	50	29	0.159
Specialist	1	1	0	5	2	
Consultant	3	2	10	11	2	
Postgraduate trainee year						
PGY^a^ 1/2	2	7	11	20	16	0.909
PGY 3/4	2	7	12	29	13	
PGY 5–7	0	0	1	1	0	
Years of experience						
2–5	0	1	0	1	1	0.393
5–10	1	0	1	5	0	
more than 10	3	2	9	11	4	
The primary site of practice						
Hamad General H^b^	8	15	31	60	32	0.515
Al Wakra H	0	0	0	2	2	
Al Khor H	0	2	3	5	0	

**Notes:** ^a^Postgraduate trainee year ^b^Hospital.

### Reported Barriers and Facilitators for Breast Cancer Screening

Unclear pathway (40%), lack of time in clinic/ward rounds (26.5%), and patient refusal (12.3%) were the major reported barriers for cancer screening. There were no statistically significant differences between those barriers when the participants were stratified according to their position, training level, or years of experience. Conversely, gender appears to influence the reporting of barriers to outpatient breast cancer screening, with a higher proportion of screening barriers due to unclear pathways reported amongst male rather than female doctors (P = 0.003) as shown in Table 8.

**Table 8:** Barriers and Facilitators of Screening

	Frequency	Percentage
Barriers to recommending breast cancer screening		
Unclear pathway	62	40.0
Not my role	14	9.0
Not sure what test to order	5	3.2
Patient refusal	19	12.3
No time in clinic/ward rounds	41	26.5
Other	14	9.0
What are the facilitators to you recommending breast cancer screening		
To be done by a nurse	17	10.7
An easy and clear pathway	74	46.5
More orientation to guidelines	47	29.6
To be done by a female doctor	18	11.3
Other	3	1.9
Who do you think should screen asymptomatic patients for breast cancer		
Nurse	6	3.8
Cancer screening program team	92	58.2
Trainees	24	15.2
Specialists/Consultants	26	16.5
Other	10	6.3

Almost half of the survey participants (46.5%) pointed out that an easy and clear pathway would facilitate breast cancer screening, while 29.6% stated that more orientation to guidelines is required. Most physicians believe that a dedicated cancer screening team should be responsible for screening the eligible patients (Table 9).

**Table 9:** Response to Whom Should Screen the Patients

	Nurse	Screening Program Team	Trainee	Specialist/Consultant	Others	P-value
Your current position						
Trainee	5	73	23	13	5	0.009
Specialist	1	4	0	3	1	
Consultant	0	14	1	9	4	
Postgraduate trainee year						
PGY^a^ 1/2	2	35	11	5	3	0.851
PGY3/4	3	37	12	7	2	
PGY5-7	0	1	0	1	0	
Years of experience						
2–5	0	1	0	1	1	0.268
5–10	0	1	1	4	1	
more than 10	1	17	0	8	3	
Primary site of practice						
HGH	6	83	24	22	9	0.757
Al Wakra H^b^	0	3	0	1	0	
Al Khor H	0	6	0	3	1	

**Notes:** ^a^Postgraduate trainee year ^b^Hospital.

## Discussion

Our survey mainly targeted postgraduate trainees as they constituted almost 76% of the study participants. The rest of the respondents were specialists and consultants. The trainees form an integral part of the governmental healthcare system and their participation in the survey was crucial.

The healthcare system in the state of Qatar is divided into governmental and private sectors. The former constitutes the major portion.^10^ The cancer screening programs in Qatar are offered only in governmental institutes where Hamad Medical Corporation (HMC) involving 12 healthcare facilities, almost makes the entire system,^11^,^12^ the other being three major primary health-care centers (PHCC). The survey was conducted in three main general hospitals in HMC, which are Hamad General Hospital, Al Wakra Hospital, and Al Khor Hospital. These hospitals cater to a major bulk of the population in Qatar.^13^ Once the physician decides to refer an eligible patient for breast cancer screening from the above-mentioned hospitals, the patient will be directed to the only specialized cancer center in Qatar named the National Center for Cancer Care and Research (NCCCR), which is again part of HMC^11^ or one of the three major PHCCs chosen for cancer screening. Patients can also book direct appointments for cancer screening at the PHCCs, but the awareness of this pathway among the general population is low.

The hospitals in HMC, both the general and specialized centers, have the trainees as the major manpower. The outpatient and inpatient care depend on the trainees with either direct or indirect supervision.^14^

The general medicine department at HMC holds a unique place in the healthcare system in Qatar in terms of the vast service area it covers and its academic contribution in teaching one of the largest residency programs. HMC is an ACGME-I (Accreditation Council for Graduate Medical Education-International) accredited institution with residents and faculty joining from many parts of the world.^14^ HMC medical education follows the North American medical education system, with internal medicine residents undertaking annual in-training exams conducted by the American College of Physicians (ACP). Due to these factors, medical professionals are better versed in international practice than on the Qatari guidelines. These subtle differences are remedied via informal intrinsic knowledge passed on by other experienced professionals.

Breast cancer epidemiology in the state of Qatar differs from other parts of the world. The median age of breast cancer incidence among women in Qatar is 47 years, compared to 63 years among the western population.^15^,^16^ This is close to the published epidemiological data from neighborhood countries in the Gulf region. In Saudi Arabia, for example, the percentage of women who are diagnosed with breast cancer below the age of 50 is 57.5%.^17^ This may indicate that women in Arabian Peninsula share similar genetic risks that are responsible for the diagnosis of breast cancer at a younger age. It is known that BRCA mutations are prevalent among Qatari breast cancer patients, reaching approximately 10%.^18^

In the era of personalized medicine, it is very important to have an awareness of local statistics and guidelines to provide appropriate care to patients. The benefits of screening mammography among women extend beyond mortality reduction to involve surgical aspects. With adherence to the screening program, it is expected to achieve better surgical outcomes in terms of higher rates of breast-conserving surgeries, and shorter hospital stay.^19^ It is also noted that women with more aggressive and rapidly growing tumors are more likely to benefit from screening mammography than other groups.^20^

Among the various options available for breast cancer screening, mammography is the best-studied and the only proven breast cancer screening modality. Its already been established that an organized population-based screening mammography program can reduce breast cancer mortality.^21^ The United States Preventive Service Task Force (USPSTF) recommends biennial screening mammography for women aged 50 to 74.^22^ Qatar national cancer screening guidelines recommend initiation of screening at a younger age due to the relatively younger demographics prevalent in the country and the earlier presentation of breast cancer. The national screening program recommends mammography to screen women between 45 and 69 years of age, with three-yearly recall.^23^

The results of this study suggested a low compliance rate with screening protocols in Qatar. Our study showed that physicians do not regularly request or advise patients for breast cancer screening. Although three-fourth of the survey participants believed that breast cancer screening should be recommended in general, the proportion of physicians recommending the screening test is 7.5% and 5% in clinic and inpatients settings, respectively. These numbers correlate well with previous compliance rates in the published literature about cancer screening.^24–26^ This result could significantly affect the number of screened patients since discussing the cancer screening process with the physician can make the patient at least four times more likely to get a mammogram.^27^ Another opportunity to offer the screening test while a patient is admitted to the hospital as it provides the physician with the chance to assess the patient’s willingness to be involved in the screening program.

The gap in the knowledge of physicians on breast cancer screening guidelines is a major concern addressed in this study. Our survey showed that around 75% of the participants would recommend screening asymptomatic patients for breast cancer, but over 50% would recommend the mammography to the wrong age group. Almost equal proportions of participants answered the question about the age group based on the local Qatari guidelines (23.8%) and the USPSTF recommendations (24.4%). The same results were seen in the interval of mammograms. Approximately 32% recommended doing mammography every two years, which follows the USPTF, and 31% recommended having breast screening every three years, which follows the local Qatari guidelines. Considering these results, we would recommend encouraging trainees and faculty to be more familiar with the local guidelines. It should also be noted that trainees were more likely to follow the correct screening protocol than faculty, which could be because most of the faculty who participated in the survey were hospitalists who do not have outpatient services.

The current internal medicine residency program does have oncology outpatient rotations as the trainees are mainly involved in inpatient services. The recommended solution is to include outpatient duties in the oncology rotation, which would improve cancer screening knowledge. One of the measures to improve compliance with cancer screening by physicians is to have flyers and posters to remind the physicians to recommend cancer screening and encourage them to ask the physician for it. Another solution is to have a reminder on the electronic medical charts that appears to the physician if the patient’s age falls within the recommended group for screening. Indeed, our survey findings were consistent with the previously published reports showing that the higher the trainees’ level, the more likely they are to offer appropriate screening tests.^28^,^29^

The participants were asked about the potential barriers to assess the reasons for the low compliance rate. We found that 40% of them reported that an unclear electronic pathway was the major obstacle as it was recently started in 2015. This is supported by the fact that only 54.9% of the survey respondents were aware of the referral pathway in the electronic medical records platform. The survey also showed that more than half of the physicians (58%, n=92) think that screening should be done by a specialized cancer screening team.

Since the launch of the National Cancer Strategy in 2011, the Qatari government has greatly supported cancer screening.^30^ Primary healthcare-based breast and bowel cancer screening has been made available throughout the country to implement the National Cancer Screening program. The electronic medical record system was modified to include pathways for referrals. Unfortunately, the trainees and senior physicians were not instructed or made aware of it, making it mostly unused.

The major strength of this study is the novelty of the survey. It is the first attempt to evaluate the breast cancer screening deficit among a major training program in the Middle East with a more than 60% response rate. Nevertheless, this survey has a few limitations. First, the survey involved only three out of the 12 hospitals forming HMC and no primary health-care center was included. Second, the sample size could have been increased to involve a larger number of specialists and consultants. Third, even though barriers and facilitators were delineated at the physician level, other system-related factors were not explored in this survey.

## Conclusion

In the current era of personalized medicine, physicians should be more oriented to local guidelines to provide optimal care to their patients. While the attitude towards breast cancer screening is positive, the overall compliance with the national recommendations is sub-optimal. Further initiatives and intervention programs are required to promote the implementation of breast cancer screening in the state of Qatar. Periodic quality improvement audits could follow this survey to ensure the sustainability of physicians’ practices. We expect that this study will serve as a baseline to monitor changes over time.
